# Using Machine Learning Technique to Predict the Most Reliable Diagnostic Finding for Foreign Body Aspiration in Children: Symptoms, Chest X-ray, or Auscultation?

**DOI:** 10.7759/cureus.32461

**Published:** 2022-12-13

**Authors:** Incinur Genisol, Osman Uzunlu

**Affiliations:** 1 Pediatric Surgery, Sisli Hamidiye Etfal Training And Research Hospital, Istanbul, TUR; 2 Pediatric Surgery, Pamukkale University School of Medicine, Denizli, TUR

**Keywords:** pediatric emergency, machine learning technique, children, foreign body aspiration, bronchoscopy

## Abstract

Foreign body aspiration (FBA) is one of the most critical and life-threatening pediatric emergency situations. Prompt diagnosis in these cases is very important as they are associated with high mortality among children. When diagnosing FBA, symptoms of the patient, auscultation findings, and chest X-ray findings are usually evaluated. In this study, we conducted a retrospective analysis of all the cases involving suspicion of FBA in children under the age of 18 years who were hospitalized in the Department of Pediatric Surgery at Denizli Pamukkale University Hospital, Turkey from January 2005 to September 2020.

Instead of traditional statistical methods, we used machine learning techniques such as random forest and logistic regression to determine which finding was diagnostically the most reliable. The variables included in the analysis that were considered to be significant were as follows: symptoms, auscultation findings, chest X-ray findings, patient gender, age, location of the foreign body, and the time of admission. For the purpose of this study, we developed four different models. Model 1 included gender, age, time of admission, location, and symptoms as variables; the correct classification rate of the model was found to be 82.3%. Model 2 included auscultation findings in addition to Model 1, and the correct classification rate of the model was 84.8%. Model 3 included chest X-ray findings in addition to Model 1, and the correct classification rate of the model was 87.4%. Model 4, on the other hand, included both auscultation findings and chest X-ray findings in addition to Model 1, and the correct classification rate of the model was 87.6%. Based on our findings, a definitive diagnosis of FBA using only symptoms, auscultation findings, or chest X-ray findings in isolation does not seem possible. Additionally, using only symptoms and chest X-ray findings is also insufficient to make a diagnosis.

## Introduction

Foreign body aspiration (FBA) is a life-threatening emergency and requires prompt diagnosis and treatment. It is frequently seen in children younger than three years of age with a peak incidence between the first and second years of life [1-3].

FBA should be suspected in any pediatric patient with respiratory symptoms since it is the fourth leading cause of death in the preschool age group as well as among adolescents [4]. Patients may not always manifest the classic triad of wheezing, coughing, and choking [5]. The presentation of the symptoms of FBA depends on the degree of airway obstruction, the location and the type of the object, as well as the age of the child, and the time elapsed since the event [6]. While clinical history and physical examination may offer some clues, FBA can only be conclusively diagnosed with bronchoscopy. Rigid bronchoscopy under general anesthesia still provides a safe and effective treatment [7].

When a child presents to the emergency department due to FBA, the patient's history is taken at the outset and the auscultation findings are evaluated. After that, the patient's chest X-ray is evaluated and, finally, the patient is quickly diagnosed based on all the obtained data. On the other hand, when the data is entered into the machine learning technique, it generates a result that helps us to make a preliminary diagnosis. It shows which findings may be more important in a set of several pertinent findings. Therefore, in this study, we aimed to identify which finding is more statistically important in the diagnosis of FBA by using the machine learning technique.

## Materials and methods

Study design and data collection

This was a retrospective, single-center, cross-sectional, descriptive study involving children aged 0-18 years who had undergone rigid bronchoscopy with suspected FBA at the Pamukkale University Hospital Pediatric Emergency Department between January 2005 and September 2020. Pamukkale University Hospital is a tertiary referral hospital with 857 beds and a considerable number of patient admissions annually.

Demographic data (age, gender), time of admission (days), presence of airway symptoms, the duration from the suspicion of FBA to hospital admission (symptomatic/asymptomatic), auscultation findings (equal/not equal for both lungs), chest X-ray findings (normal/abnormal), bronchoscopy results (foreign body location, the structure of the foreign body), and duration of hospitalization (days) were collected from the records in the hospital information management system. Since there was no flexible bronchoscope for children in our hospital, all children underwent rigid bronchoscopy under general anesthesia by a pediatric surgeon.

Four different models were created to evaluate the efficacy of symptoms, auscultation, and chest X-ray findings in the diagnosis of FBA (Table 1).

**Table 1:** Representation of the data used in the four models created

Models	Gender	Age	Time of admission	Location of the foreign body	Symptom	Auscultation	Chest X-ray
Model 1	+	+	+	+	+	-	-
Model 2	+	+	+	+	+	+	-
Model 3	+	+	+	+	+	-	+
Model 4	+	+	+	+	+	+	+

Machine learning techniques such as logistic regression, naive Bayes, support vector machine, bagging, and random forest were used based on the performance criteria of accuracy, F-measure, the area under the receiver operating characteristic (ROC) curve (AUC) (ROC area), and precision-recall curve (PRC) area (PRC area) to evaluate the prediction performances of the models.

In our results, we used the machine learning technique, which is a model that can speed up the decision-making process, instead of traditional statistics. Machine learning techniques show great promise in the field of diagnosis in healthcare.

Statistical analysis

SPSS Statistics v. 11.5 (IBM Corp., Armonk, NY) program was used in the analysis of the data. Mean ±standard deviation (SD) and median (min-max) were used for quantitative variables, and the number of patients (percentage) was used for qualitative variables. The Mann-Whitney U test was used to analyze continuous variables between groups according to the distribution of the normality of the variables. Chi-square and Fisher's exact tests were used to evaluate the relationship between two qualitative variables. Variables were analyzed at a 95% confidence interval and p<0.05 was considered statistically significant. Classification methods of logistic regression and random forest were used in the WEKA program. The data set was evaluated using the 10-fold cross-validation test option. Accuracy, F-measure, PRC area, and ROC area were used as machine learning performance criteria.

## Results

Between January 2005 and September 2020, rigid bronchoscopy was performed in 236 children with suspicion of FBA. Five children who lacked follow-up data were excluded from the study. Finally, 231 children were included in the study. The mean age of the subjects was 28.2 ±27.0 months and 153 (66.2%) of the children were male.

The mean admission time was 3.9 ±11.8 days. A total of 77 (33.3%) children had no symptoms of airway obstruction. Of the children included in the study, 62.3% had abnormal auscultation findings and 51.9% had abnormal chest X-ray findings. The foreign body was located in the left lung in 55.6% of the children, in the right lung in 35.2%, and in the trachea in 9.2%. A summary of the demographic data is presented in Table 2.

**Table 2:** Demographic data SD: standard deviation

Variables		Values
Gender, n (%)	Male	153 (66.2)
	Female	78 (33.8)
Age, months	Mean ±SD	28.2 ±27.0
	Median (min-max)	21.0 (5.0-192.0)
Age, years, n (%)	<1	22 (9.5)
	1-2	121 (52.4)
	2-3	58 (25.1)
	3-4	4 (1.7)
	4-5	4 (1.7)
	5-6	8 (3.5)
	>6	14 (6.1)
Symptom, n (%)	Symptomatic	154 (66.7)
	Asymptomatic	77 (33.3)
Duration from symptom finding to time of admission (days)	Mean ±SD	3.9 ±11.8
	Median (min-max)	1.0 (0.0-150.0)
Auscultation findings, n (%)	Equal for both lungs	87 (37.7)
	Not equal	144 (62.3)
Chest X-ray findings, n (%)	Normal	111 (48.1)
	Abnormal	120 (51.9)
Location of foreign body, n (%)	Right lung	69 (35.2)
	Left lung	109 (55.6)
	Trachea	18 (9.2)
Foreign body, n (%)	Not found	34 (14.7)
	Found	197 (85.3)
Structure of foreign body, n (% of found foreign bodies)	Inedible	27 (13.7)
	Edible	170 (86.3)
Length of hospitalization, days	Mean ±SD	2.8 ±2.2
	Median (min-max)	2.0 (0.0-13.0)

**Figure 1:**
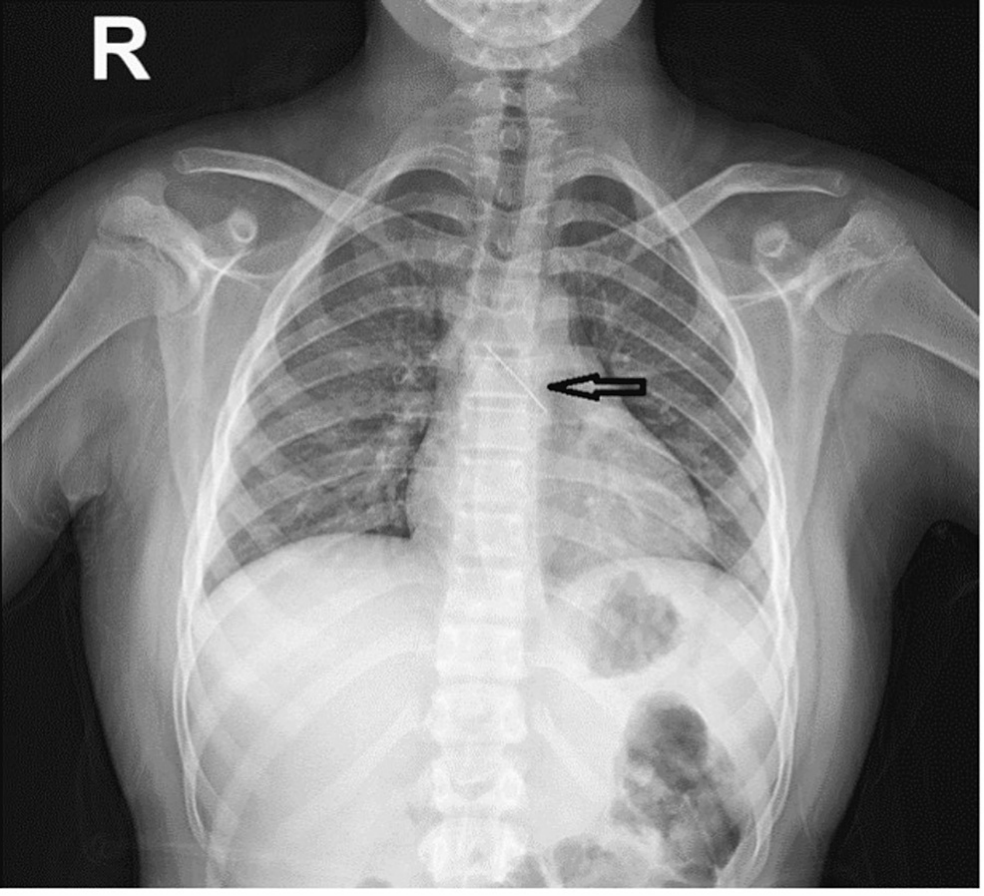
Chest radiography of one of the patients before bronchoscopy - turban pin is in the left main bronchus

A foreign body was found in 197 children. Of them, 170 children (86.3%) had edible objects, as follows: peanut (n=48; 24.4%), hazelnut (n=22; 11.2%), crackers (n=11; 5.6%), and watermelon (n=2; 1.0%). Inedible foreign bodies were found in 27 (13.7%) patients. The most commonly removed inedible objects were seeds (n=12; 6.1%), plastic pieces (n=7, 3.6%), and turban needles (n=5; 2.5%). One of the chest radiographs demonstrating the turban needle is depicted in Figure 1. All the patients who aspirated turban scarf needles were adolescent girls with a mean age of 12.4 ±2.0 years.

The foreign body was found more frequently in children with abnormal auscultation and chest X-ray findings (p<0.001 and p=0.013, respectively). However, age, gender, time of admission, and length of hospitalization did not differ according to the presence of the foreign body (Table 3).

**Table 3:** Association of demographic, clinical, and radiological findings with the presence of foreign body ^a^Chi-square test. ^b^Fisher's exact test. ^c^Mann-Whitney U test SD: standard deviation

Variables		Foreign body		
		Not found	Found	P-value
Gender, n (%)	Male	24 (70.6)	129 (65.5)	0.561^a^
	Female	10 (29.4)	68 (34.5)	
Age, months	Mean ±SD	30.1 ±29.4	27.9 ±26.7	0.605^c^
	Median (min-max)	18.5 (6.0-156.0)	21.0 (5.0-192.0)	
Age, years, n (%)	<1	4 (11.8)	18 (9.1)	0.064^b^
	1-2	19 (55.8)	102 (51.8)	
	2-3	4 (11.8)	54 (27.4)	
	3-4	0 (0.0)	4 (2.0)	
	4-5	1 (2.9)	3 (1.5)	
	5-6	4 (11.8)	4 (2.0)	
	>6	2 (5.9)	12 (6.1)	
Symptom, n (%)	Symptomatic	31 (20.1)	123 (79.9)	0.001^a^
	Asymptomatic	3 (3.9)	74 (96.1)	
Time of admission (time from the onset of suspected aspiration or symptoms to admission to the hospital)	Mean ±SD	6.1 ±25.5	3.6 ±7.3	0.060^c^
	Median (min-max)	1.0 (0.0-150.0)	1.0 (0.0-90.0)	
Auscultation findings, n (%)	Equal for both lungs	22 (25.3)	65 (74.7)	<0.001^a^
	Not equal	12 (8.3)	132 (91.7)	
Chest X-ray findings, n (%)	Normal	23 (20.7)	88 (9.3)	0.013^a^
	Abnormal	11 (9.2)	109 (90.8)	
Length of hospitalization (days)	Mean ±SD	3.3 ±3.0	2.7 ±2.0	0.897^c^
	Median (min-max)	2.0 (0.0-13.0)	2.0 (0.0-11.0)	

There was no statistically significant difference between symptomatic and asymptomatic children in terms of the location of the foreign body (p=0.318, Table 4). Of note, 63.5% of the patients who aspirated edible foreign bodies and 55.6% of the patients who aspirated inedible foreign bodies were symptomatic, which revealed a non-significant association (p=0.427, Table 4).

**Table 4:** Association between symptoms and the structure of the foreign body ^a^Chi-square test. ^b^Fisher's exact test. ^c^Mann-Whitney U test SD: standard deviation

Variables		Symptoms		
		Symptomatic	Asymptomatic	P-value
Age, months	Mean ±SD	28.2 ±27.8	28.3 ±25.5	0.484^c^
	Median (min-max)	20.0 (6.0-192.0)	22.0 (5.0-132.0)	
Age, years, n (%)	<1	15 (9.7)	7 (9.1)	0.572^b^
	1-2	82 (53.3)	39 (50.6)	
	2-3	35 (22.8)	23 (29.9)	
	3-4	3 (1.9)	1 (1.3)	
	4-5	4 (2.6)	0 (0.0)	
	5-6	7 (4.5)	1 (1.3)	
	>6	8 (5.2)	6 (7.8)	
Location of the foreign body, n (%)	Right lung	48 (39.0)	22 (29.7)	0.290^a^
	Left lung	66 (53.7)	43 (58.1)	
	Trachea	9 (7.3)	9 (12.2)	
Structure of the foreign body, n (%)	Inedible	15 (9.7)	12 (15.6)	0.003^a^
	Edible	108 (70.2)	62 (80.5)	
	Nonidentified	31 (20.1)	3 (3.9)	

In addition, the association of the structure of the object with auscultation findings and chest X-ray findings was found to be significantly different (p=0.001 and p=0.012, respectively). While 55.6% of the patients with inedible foreign bodies had abnormal auscultation findings, 68.8% of the patients with edible foreign bodies had abnormal findings. While 32.4% of the patients with inedible foreign bodies had normal chest X-rays, 57.6% of the patients with edible foreign bodies had normal X-rays.

**Figure 2:**
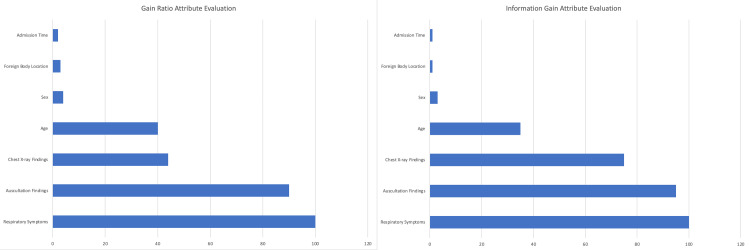
Significance of variables regarding the presence of the foreign body

As shown in Figure 2, the importance of the variables and the value that the variables add to the data set for the presence of a foreign body were examined by using gain ratio and information gain attribute evaluation tests. The order of significance of the variables included in the analysis, which are considered to be statistically and clinically significant in the study, is as follows: symptoms, auscultation findings, chest X-ray findings, age, sex, foreign body location, and time of admission. Feature selection is a method of filtering out the important features as all the features present in the dataset are not equally important. There are some features that have no effect on the output, and hence we can skip them since our motive is to narrow down the data before feeding it to the training model. Hence, feature selection is performed before training machine learning models. We used the variables presented in Figure 2 in this study.

Various machine learning techniques such as logistic regression, naive Bayes, support vector machine, bagging, and random forest were used to evaluate the prediction performances of the models. The results of the logistic regression and random forest methods, which are the methods that give the best results in each model, are given in Table 5. When the performance criteria of accuracy, F-measure, ROC area, and PRC area are evaluated together, the logistic regression method gives the best results as seen in the table. According to this method, when the variables of gender, age, time of admission, location, and symptoms were evaluated together, the correct classification rate of the model was found to be 82.3%. In other words, when we make an estimation about the presence/absence of a foreign body with this model, the accuracy rate of this estimation is 82.3%. Specifically, if the estimation of the presence/absence of the foreign body is made with this model for 100 patients who were admitted to the clinic, the estimation regarding approximately 83 patients will be correct. While the correct classification rate of model 2 with the logistic regression method was found to be 84.8%, that of model 3 with the logistic regression method was found to be 87.4%, and that of model 4 with the logistic regression method was found to be 87.6%.

**Table 5:** Machine learning technique results of the models ROC: receiver operating characteristic; PRC: precision-recall curve

Models	Methods	Accuracy	F-measure	ROC area	PRC area
Model 1	Logistic regression	0.823	0.787	0.782	0.857
	Random forest	0.814	0.791	0.620	0.806
Model 2	Logistic regression	0.848	0.841	0.824	0.874
	Random forest	0.801	0.774	0.646	0.805
Model 3	Logistic regression	0.874	0.868	0.821	0.872
	Random forest	0.831	0.810	0.680	0.815
Model 4	Logistic regression	0.876	0.869	0.833	0.876
	Random forest	0.818	0.798	0.732	0.846

When the four created models were evaluated according to the logistic regression method, it was observed that the accuracy rate increased from model 1 to model 4 (82.3% for model 1 and 87.6% for model 4; Table 5). When the models are examined, the rate of detecting the presence of the foreign body in auscultation findings in addition to the symptoms increases by 2.5%. When a chest X-ray is evaluated in addition to the symptoms, the rate of foreign body detection increases by 5.1%. When auscultation and chest X-rays are evaluated in addition to the symptoms, the rate of foreign body detection increases by 5.3%.

## Discussion

It is very difficult to diagnose FBAs in pediatric patients that occur without an eyewitness present. Moreover, rigid bronchoscopy is not a simple surgical procedure. For this reason, FBA in children is an issue that must be always held in mind and kept on the agenda. When we retrospectively screened the patients who underwent rigid bronchoscopy at our clinic in order to facilitate the diagnosis, we determined that the patient being symptomatic is the most valuable indicator of FBA.

FBAs usually occur in infants (mean age of 28.2 months in our series) with a male-to-female ratio of 2:1 as per the literature [8], which aligns with our findings as well. The most common types of aspirated foreign bodies are organic substances [9]. Peanut is reported to be the most common type of foreign body, which is consistent with our results. Turban scarf needle aspiration is common in adolescent girls aged 10-16 years in our country [10,11]. In Muslim societies such as ours, adolescent girls hold the needle between their lips while wearing a scarf and can aspirate the needle while talking or laughing. This has been recently referred to as "scarf pin-related Hijab syndrome" in the literature [11]. In our series, turban needles were removed from five adolescent girls.

When the data of symptomatic and asymptomatic patients were compared, we found that they differed only with regard to the structure of the foreign body. A higher rate of edible foreign bodies was seen in symptomatic patients. A similar association was also seen regarding chest X-rays and auscultation findings of the patients. It was thought that an edible foreign body may cause more symptoms since it has a softer surface, is digestible, swells when wet, and can obstruct the bronchus.

A study by Zhu et al. reported that FBAs were misdiagnosed at a rate of 35.6% in healthcare institutions other than tertiary care centers [12]. They showed that the most common misdiagnosis was bronchiolitis (51.3%) [12]. Keeping this in mind, we aimed to achieve more accurate results by scanning previous patient records and trying different models to prevent the physician who first saw the patient from making a wrong diagnosis. The most important diagnostic clue in suspected FBA is the history of witnessed aspiration in children; however, it may not be always present in FBA cases [13-15].

In the literature, it has been shown that chest X-ray is not as reliable as CT in diagnosing foreign bodies in adult patients [16]. However, we normally avoid performing CT in children because of the intense amount of ionizing radiation and the need for sedative agents to avoid motion-related artifacts. We believe that the new scoring systems will help diagnose foreign bodies more accurately [17]. 

Our study has a few limitations. We conducted our study at a tertiary care hospital. Hence, our findings may not be applicable to patients treated at non-tertiary centers. Another limitation is that we collected our data from a single healthcare center. And finally, we recognize that we cannot fully guarantee the accuracy of the information in the available health records, and it could limit our findings as well.

## Conclusions

In most cases, the event of FBAs in children occurs unwitnessed. Hence, the family members usually do not remember most of the symptoms at the time of presentation or cannot fully describe them when they arrive at the hospital. Hence, clinicians should always suspect FBA when a child presents with respiratory symptoms. A chest X-ray alone without auscultation is insufficient to confirm FBA in pediatric patients, irrespective of whether respiratory symptoms are present or not. Therefore, as done routinely in most of the clinics, all patients with suspected FBA should be questioned about symptoms at the outset; auscultation should be performed as part of the physical examination, and a chest X-ray should be requested.
